# Prediction of Quality of Life in Asian Patients with Schizophrenia: A Cross-sectional Pilot Study

**DOI:** 10.3389/fpsyt.2017.00198

**Published:** 2017-10-05

**Authors:** Carol C. Choo, Peter K. H. Chew, Cyrus S. Ho, Roger C. Ho

**Affiliations:** ^1^College of Healthcare Sciences, James Cook University Singapore, Singapore, Singapore; ^2^Department of Psychological Medicine, National University of Singapore, Singapore, Singapore; ^3^National University Health System, Singapore, Singapore

**Keywords:** schizophrenia, quality of life, coping, psychosocial, predictors

## Abstract

**Background:**

There has been a shift in mental health services from an emphasis on treatment focused on reducing symptoms to a more holistic approach involving quality of life (QOL) and overall well-being. Many psychosocial variables are associated with QOL but a parsimonious framework is needed to deepen our understanding about the contribution of psychosocial factors in influencing the QOL of Asian patients with schizophrenia in Singapore. The study aimed to address the current gap in literature by analysis of QOL using available predictors in Asian patients with schizophrenia in Singapore.

**Methods:**

43 Singaporean patients diagnosed with schizophrenia were recruited at a large teaching hospital in Singapore from January to May 2010 and were invited to complete questionnaires. Of the sample, 65.1% were females, ages ranged from 18 to 65 (M = 44.60, SD = 12.19). Available variables were subjected to regression analysis.

**Findings:**

The data were analyzed using SPSS Version 23 with the alpha level set at 0.05. The final model with five predictors was significant in predicting QOL. Positive Re-appraisal, Social Support, Avoidant Coping, Duration of Hospitalization, and Education accounted for 47.2% of the variance (Adjusted *R*^2^ = 40.0%) in QOL, *F* (5, 37) = 6.60, *p* < 0.001. Those with post-secondary or higher education had higher QOL than those with secondary or lower education. Duration of hospitalization negatively predicted QOL.

**Conclusion:**

The findings were discussed in regards to clinical implications for informing interventions to enhance QOL in patients with schizophrenia.

## Introduction

There has been a shift in mental health services from an emphasis on treatment focused on reducing symptoms, based on a narrow conceptualization of health and disease, to a more holistic approach which considers quality of life (QOL), well-being, and overall functioning (1). QOL is an increasingly critical outcome of mental health care (2). Lower QOL has been associated with mental disorders, such as schizophrenia (2, 3). Schizophrenia is a chronic disorder characterized by its debilitating course (4). Functional impairment is high, leading to lost wages and work impairment, with related personal, societal, and economic burdens (5, 6). More mental health services are adopting the recovery paradigm (7), focusing on rehabilitation (8). Recovery is multi-faceted (9), it encompasses hopefulness and empowerment and the pursuit of life goals, aspirations, and valued social roles (10). Recovery is also associated with resilience, coping, better management of symptoms, decreased risk of hospitalization (11), and enhanced social functioning (12) which are related to QOL.

Considerable variations have been observed in the clinical course and outcome of schizophrenia between Western and Asian countries and also within Asian countries, but reasons underlying these variations remain unexplored although it is suggested that cultural explanations may provide clues (13). Research is much needed in the local Asian population in Singapore as it cannot be assumed that research findings conducted in other countries would also be relevant to the local context. Discrepancies are seen between Asian and Western samples, e.g., functional support concerning quality of relationships and perceived social support were found to be related to QOL in Western samples (14, 15) but not in Asian samples (8). On the other hand, self- efficacy was associated with lowered stress and enhanced mental health and QOL in Western samples (16, 17), as well as QOL in Asian samples (18, 19). Recent research in Singapore using Asian samples suggests that psychosocial variables, e.g., higher education level, contribute to QOL (20). Other recent Asian studies suggest that shorter duration of hospitalization (21) is prominent in promoting QOL (22), together with possible influences from demographic variables, such as gender (21, 23) and age (7). Although a recent review suggests that gender may not emerge as a significant variable (9), recent large scale studies in Asian samples in Singapore indicate that demographic variables, e.g., age (24) and gender (25), need to be considered for possible influences on the overall wellbeing of Singaporeans. Relevant research suggests that there might be commonalities between research done in Western and Asian samples in the contribution of psychosocial factors in QOL, but cultural influences may affect the prominence of certain unique variables, and it is yet unclear how these might vary specifically in the Asian population in Singapore. The aforementioned highlights the need for a coherent and parsimonious framework to understand the contribution of various psychosocial factors to QOL relevant to patients with schizophrenia for the Asian population in Singapore.

Besides the aforementioned variables, another body of research looks into coping and QOL. Individuals with mental illness, e.g., schizophrenia (26), tend to report lower distress if they employ adaptive coping strategies (26). Usage of a repertoire of more than one adaptive coping strategy leads to greater effectiveness in managing psychotic symptoms. Literature suggests that individuals with schizophrenia tend to use suppression as an emotion regulation strategy rather than more adaptive strategies, e.g., re-appraisal, often used by healthy controls (27). Maladaptive coping strategies may adversely disturb the overall functioning of individuals with mental disorders (28) and add to caregiver burden as well as various personal and societal burdens (6). The mechanisms determining choice and efficacy of coping strategy for individuals with schizophrenia remain unclear (26). It is also unclear if there are cultural influences on coping and QOL for individuals with schizophrenia.

This study aims to address the current gap in literature by examining the ability of available variables namely demographic characteristics, such as age, gender, and education, and psychosocial variables namely functional social support, self-efficacy, and coping styles, as well as duration of hospitalization in predicting QOL of Asian patients with schizophrenia in Singapore. It is hypothesized that more than one style of coping (29), more social support (22), and higher education (20) would positively predict QOL, while duration of hospitalization (21) would negatively predict QOL. There might be possible influences from demographic variables, such as gender (21, 23) and age (7).

## Materials and Methods

### Procedure

Ethics approval was obtained from the Domains-Specific Review Board of a large teaching hospital in Singapore and the James Cook University Human Research Ethics Committee. 43 patients who were Singaporean citizens and permanent residents with a diagnosis of schizophrenia were recruited from an outpatient clinic of a large teaching hospital in Singapore from January to May 2010. Eligibility criteria for the study included the following: (a) above the legal age of consent, i.e., aged 21 years and above; (b) able to understand and respond to questions in English and/or Mandarin; and (c) absence of intellectual impairment. The patients’ subjective ratings on QOL, social support, and coping style were investigated with the use of a paper–pencil self-report survey.

Individuals who met the inclusion criteria were approached and briefed by hospital clinical staff regarding the objectives of the study. Individuals who agreed to be part of the study were then asked to sign a consent form, and they were given the questionnaires thereafter. To decrease social desirability bias, participants were told that their responses would be kept confidential, and they were assured that there were no right or wrong answers to the questionnaire items. After completion of the questionnaire, participants were subsequently debriefed and thanked for the time spent in this study.

There were a total of 43 participants (65.1% females) with schizophrenia in the study. Their age ranged from 18 to 65 (M = 44.60, SD = 12.19). The characteristics of the sample are presented in Table 1.

**Table 1:** Characteristics of the sample (*n* = 43).

	*n*	%		
Education				
Secondary school or lower	29	67.4		
Post-secondary or higher	14	32.6		
Marital status				
Single	26	60.5		
Married/divorced/separated	17	39.5		
Employment				
Employed	18	41.9		
Unemployed	25	58.1		

	**M**	**SD**	**Min.**	**Max.**

Duration of (in days)				
Schizophrenia	133.34	128.19	1	480
Antipsychotic treatment	33.67	91.18	0	360
Hospitalization (over past year)	0.56	0.63	0	2
Overall Schizophrenia Severity	23.42	6.78	–	–

### Materials

The four-page questionnaires included items on: demographic background, namely, age, gender, and education, and the following questionnaires. QOL was measured using the 12-Item Short Form Health Status Questionnaire [SF-12 (30)]. Eight domains of health which included physical functioning, role limitations due to physical and emotional problems, bodily pain, general health, vitality, social functioning, and mental health were assessed across 12 items. Higher scores denote better perceived QOL. High test/retest validity as well as good internal reliability has been derived with Asian populations (31). Level of functional social support was assessed by a four-item measure designed to detect various archetypal functions of informational support, emotional support, positive social interaction (32), and ability to promote self-disclosure (33). This measure is scored on a 5-point Likert scale (1 = strongly agree and 5 = strongly disagree). Scores range from 4 to 20. Higher overall scores show perception of greater support from spouses, families and/or friends. The internal reliability was 0.85 (34). Self-efficacy was measured using the Self Mastery Scale (SMS) (35). The SMS consists of seven items scored on 5-point Likert Scale (1 = strongly and 5 = strongly disagree). Scores range from 7 to 35. Higher overall scores show greater tendency to perceive life events as under self-control rather than that of external forces. High face validity and good internal reliability were demonstrated in Asian populations (36). The severity of the schizophrenia was assessed using the Brief Psychiatric Rating Scale (BPRS) (37). The scale consists of 18 items rated on 7-point scale of severity (1 = *not present* and 7 = *extremely severe*), based on behavioral anchors, with a highest possible score of 126. An overall measure of symptom severity is obtained by summing up the severity scores of all 18 items. The BPRS has been used with Asian populations (38). Coping styles were self-reports measured using 5-point Likert scales on 4 items, specifically the coping styles detailed below, regarding the extent to which patients agreed with the statements on how they tried to cope with stressors that they have faced in their lives (1 = *strongly disagree* and 5 = *strongly agree*). Scores range from 1 to 5. Higher item scores show greater agreement in using the respective coping style. The coping styles were active problem coping (planning and taking steps to deal with the stressors), positive re-appraisal (changing to more optimistic and positive perception) seeking social support, and escape-avoidance coping or avoiding dealing with the problems. The self-report was developed for the purpose of the current study and based on previous literature on coping (39, 40). Psychometric properties for usage of the above self-report in Asian populations are not currently available.

### Data Analysis

The data were analyzed using SPSS Version 23 with the alpha level set at 0.05. Stepwise regression was used to assess the ability of the predictor variables to predict QOL. Preliminary analyses were conducted to ensure no violation of the assumptions of normality, linearity, homoscedasticity, and multicollinearity. These predictor variables included: age, gender (males versus females), education (secondary school or lower versus post-secondary or higher), marital status (single versus married or divorced or separated), employment (employed versus unemployed), duration of schizophrenia, duration of antipsychotic treatment, duration of hospitalization, overall schizophrenia severity, social support, self-efficacy, problem coping, positive re-appraisal, support coping, and avoidant coping. To arrive at a parsimonious model for the prediction of QOL, the available variables were entered into the regression analysis model, and stepwise procedure was used to condense the variables. Variables were eliminated from the model based on likelihood ratio tests. This method of stepwise regression analysis was found to be appropriate in similar studies to analyze contribution of large numbers of available scale and ordinal variables into a parsimonious statistical prediction model (24, 25, 41).

## Results

The means and SDs for the continuous predictor variables and the criterion variable are presented in Table 2.

**Table 2:** Means, SDs, minimum and maximum scores of the variables.

	M	SD	Min	Max
Predictors				
Social support	14.58	2.74	7	19
Self-efficacy	22.49	3.08	16	31
Problem coping	3.84	0.81	2	5
Positive re-appraisal	3.47	1.03	1	5
Support coping	3.91	0.97	1	5
Avoidant coping	2.88	1.31	1	5
Criterion				
QOL	92.74	13.74	49.5	114.7

Table 3 presents the results of the stepwise multiple regression analyses used to predict QOL. Positive re-appraisal, social support, avoidant coping, duration of hospitalization, and education (primary or lower versus post-secondary or higher) accounted for 47.2% of the variance (adjusted *R*^2^ = 40.0%) in QOL, *F* (5, 37) = 6.60, *p* < 0.001.

**Table 3:** Stepwise regression with quality of life as the dependent variable.

		95% CI for B				
	*B*	Lower	Upper	Beta	*t*	*p*
Positive re-appraisal	4.96	1.57	8.35	0.37	2.97	0.005
Social support	1.29	0.03	2.54	0.26	2.08	0.044
Avoidant coping	3.37	0.76	5.98	0.32	2.62	0.013
Duration of hospitalization	−8.87	−14.71	−3.03	−0.41	−3.08	0.004
Education	8.18	0.43	15.93	0.28	2.14	0.039

## Discussion

This study aimed to explore prediction of QOL in Asian patients with schizophrenia in Singapore. As hypothesized, the model containing five predictors was significant in predicting QOL. Coping by positive re-appraisal, avoidant coping, level of functional social support, and education positively predicted QOL. Those with post-secondary or higher education had higher QOL than those with secondary or lower education. Duration of hospitalization negatively predicted QOL.

The findings are consistent with previous literature associating coping and other psychosocial and demographic variables with QOL (20, 29). The results suggest that individuals who employed two styles of coping [re-appraisal and avoidant coping], higher level of functional social support, shorter duration of hospitalization, and higher education had higher QOL. The finding is consistent with literature indicating that usage of a repertoire of more than one coping strategy leads to greater effectiveness in managing psychotic symptoms (26). The finding on functional support and QOL is consistent with previous research done in Western samples (14, 15), and contrary to a previous study done on another Asian sample (8). The finding that higher education contributes to QOL is consistent with previous studies in both Asian (20) and Western samples (42). The finding that shorter duration of hospitalization is prominent in promoting QOL is consistent with other studies (21).

Although the sample excluded patients with intellectual impairment, it might have included patients with neurocognitive and executive functioning deficits, or other psychosocial impairments that need to be further explored in future research for adverse effects on social cognition (3), as well as academic achievements and educational level which in turn affect QOL. Severity of the impairment could impact employment, coping, and ability for planning which could also affect QOL. Longer hospitalization may reflect greater severity of the illness or lack of family or community support to effectively manage the patient outside of the hospital. Most of the sample were single and about half of the sample were unemployed. It is unclear if such variables namely marital and employment status might affect functional social support or if they are reflective of the adverse psychosocial impact of the illness. Information on living conditions, e.g., if they are residing with their family or alone, is not gathered, but such factors might impact on functional social support. Comorbid illness (especially depression) is common among patients with schizophrenia, and this is likely to impact on QOL; however, such information was not collected, but needs to be considered in future research. Overall Schizophrenia Severity was included as a predictor in the current study but did not emerge as a significant predictor of QOL. This variable should be examined in more detail in future study, using a more comprehensive clinical assessment or a structured interview. Future research could seek to include patients with intellectual impairment, which were excluded in the current study.

Broad health questionnaires such as the SF-12 used in the current study might not adequately capture all factors that determine overall well-being (43). Semi-structured interviews or focus groups may uncover more in-depth information using a qualitative paradigm. It is desirable to have multi-level modeling analysis to identify different layers of factors contributing to QOL of patients with schizophrenia, which take into account the social cultural environment and other contextual factors that might impact on them, in addition to severity of psychosocial impairments of the mental illness. These factors include, e.g., socioeconomic status, ethnicity, and stigma. Future research could gather more detailed information and employ multi-level modeling analysis to give further understanding into QOL of patients with schizophrenia. In-depth interviews have been used to elicit rich perspectives (23) on coping with schizophrenia in India. Future local studies could also employ in-depth qualitative interviews to explore the relationships and processes underpinning how variables interact and impact on QOL. In this study, coping styles were assessed by self-report. The limitations are that insight and self-appraisal are needed to understand one’s own strategies; which could have impacted on findings. With the patients’ consent, family members or clinicians could be interviewed for corroboratory information. Future research could also consider using a clinician-rating scale.

The current findings have implications for community interventions, therapy and case management of patients with schizophrenia. The findings are in alignment with the recovery paradigm (7), that recovery is multi-faceted, including both clinical remission and broader social functioning (9). Clinicians could support patients’ recovery through enhanced coping, better management of symptoms (11), and social functioning (12), which are related to QOL. Therapeutic interventions could be more targeted toward enhancing coping by re-appraisal of emotionally valenced stimuli, e.g., re-interpreting a disturbing remark so it is less disturbing, a technique often used in Cognitive Behavioral Therapy (44). Interventions to enhance QOL could also target functional social support, with greater involvement of community agencies to reach out to those with schizophrenia, e.g., in support groups, or leisure groups, where individuals with schizophrenia could be encouraged to re-integrate into the community and engage in leisure activities typically used for avoidance coping. Mental health promotion could be encouraged to reduce stigma related to schizophrenia in the community and in schools, and family support could be encouraged by offering psychoeducation to family members of individuals with schizophrenia. These recommendations are consistent with suggestions made in other recent Asian studies to promote family interventions (45) and to reduce stigma (2).

Limitations to the study include the use of cross-sectional data and reliance on self-reports. Although the term prediction is used, given that the regression analysis was conducted on cross-sectional data collected at a single time-point, the direction of the relationship is a theory-derived assumption. A longitudinal design would enhance the interpretation of causality. With the patients’ consent, family members or clinicians could be interviewed for corroboratory information. The sample size is quite small, future research could replicate the study with a larger sample size of patients from polyclinics and both private and public hospitals in Singapore or consider combining data with similar research in other Asian countries. Finally, the study is limited by the predictors included in the regression model. There may have been significant predictors of QOL that were not assessed by our questionnaire and hence not included in the regression model. One such predictor may include ethnicity, a large scale recent study on Asian samples in Singapore indicated that demographic variables, e.g., ethnicity (41) need to be considered for possible influences on the overall wellbeing of Singaporeans. The major ethnic groups in Singapore include Chinese, Indian, and Malay, and local studies offer unique opportunity to examine ethnic influences and inherent cultural strengths and vulnerabilities which in turn informs targeted interventions to enhance overall wellbeing (41). Another variable not currently investigated include severity of intellectual impairment and comorbid conditions, which could be further explored in future research, due to adverse effects on social cognition (3), as well as occupational and academic achievements which in turn affect QOL.

In conclusion, the findings have implications for informing our efforts in interventions to enhance QOL for individuals with schizophrenia. By using strategies substantiated by empirical findings from current research in the local population, the clinician could be taking a step forward in utilizing the scientist–practitioner model in their evidence based practice. This study adds to the current literature in QOL for individuals with schizophrenia, and draws further focus to the importance of community management of those with mental illness. Interpreted with other research in the area, the findings are promising to inform our efforts in community management and psychosocial interventions to enhance QOL of individuals with schizophrenia, to enhance coping, family and social support, as opposed to long-term hospitalization.

## Ethics Statement

This study was carried out in accordance with the recommendations of the Domains-Specific Review Board of a large teaching hospital in Singapore, and the James Cook University Human Research Ethics Committee, with written informed consent from all subjects. All subjects gave written informed consent in accordance with the Declaration of Helsinki. The protocol was approved by the Domains-Specific Review Board of a large teaching hospital in Singapore and the James Cook University Research Ethics Committee.

## Author Contributions

RH and CH collected the patient data. RH provided access to the data and consultation. PC performed the data analysis. PC and CC interpreted the data and contributed in writing the manuscript. RH, CH, CC, and PC conceptualized the study. All authors contributed to the revisions. All authors read and approved the manuscript and agreed to be accountable for all aspects of the work.

## Conflict of Interest Statement

The authors declare that the research was conducted in the absence of any commercial or financial relationships that could be construed as a potential conflict of interest.
